# *Streptococcus bovis* Bacteremia in Neonates in a Predominantly Hispanic Population

**DOI:** 10.3389/fped.2015.00092

**Published:** 2015-10-30

**Authors:** Alicia Alvarez, Yi Jia, Cesar J. Garcia, Eduardo D. Rosas-Blum, Darius Boman, Marc J. Zuckerman

**Affiliations:** ^1^Department of Medicine, Texas Tech University Health Sciences Center, El Paso, TX, USA; ^2^Department of Pediatrics, Texas Tech University Health Sciences Center, El Paso, TX, USA; ^3^Department of Pathology, Texas Tech University Health Sciences Center, El Paso, TX, USA

**Keywords:** *Streptococcus bovis*, bacteremia, Hispanic, neonates

## Abstract

**Background:**

*Streptococcus bovis* bacteremia has been associated with gastrointestinal diseases, especially colon cancer, neoplastic colon polyps, and other malignancies of the GI tract in adults. Sporadic cases of *S. bovis* disease have also been reported in neonates and young infants. Although uncommon, *S. bovis* infection can cause fulminant neonatal sepsis and meningitis.

**Objectives:**

We report a series of pediatric patients with *S. bovis* bacteremia in a county hospital in a United States–Mexico border city in order to examine the demographic and clinical associations.

**Methods:**

We characterized the demographic and clinical features in all pediatric patients with blood cultures positive for *S. bovis* at University Medical Center in El Paso, TX, USA between January 2000 and December 2010. Hospital records were systematically reviewed by using a standardized protocol.

**Results:**

A total of seven episodes of *S. bovis* bacteremia were documented in seven pediatric patients (four female and three male). Mean age was 1.2 days (range 1–3 days), all were Hispanic, average birth weight (3.25 kg). Mode of delivery was spontaneous vaginal delivery (five) and Caesarian section (two). All of our patients developed early (<1 week) onset disease and presented with signs of respiratory distress. Five out of seven babies presented with abdominal distention and diarrhea. Six had clinical evidence of sepsis at presentation. Respiratory distress was the most common manifestation of sepsis (seven). Aspiration pneumonia was diagnosed in two of them. Most patients were treated with a combination of antibiotics (six), either ampicillin and gentamicin or ampicillin and cefotaxime, and one with ampicillin alone. None of the pediatric patients had endoscopy and none of them died.

**Conclusion:**

*Streptococcus bovis* is considered as an uncommon pathogen in the newborn, but can be associated with substantial morbidity and mortality if not identified and treated early. Physicians should be alert to the less common presentation of neonatal bacteremia due to *S. bovis*.

## Introduction

*Streptococcus bovis* is a non-enterococcal *streptococcus* in the Lancefield group D that may be found as a normal inhabitant of the human gastrointestinal tract in up to 16% of the population. The association between *S. bovis* bacteremia with endocarditis and gastrointestinal diseases, especially colon cancer, neoplastic colon polyps, and other gastrointestinal malignancies in adults has been well established in the literature (1–7). A meta-analysis reported that patients with positive *S. bovis* infection had higher concomitant rates of adenomas (43%) and colon carcinomas (18%) compared with the general asymptomatic population (8). Fernandez-Ruiz et al. reported that adenomatous polyps were found in 21/59 patients (36%) and was the most common finding among all abnormal colonic pathology results (9).

Several cases of *S. bovis* bacteremia have been reported in neonates and young infants (10–14). The clinical conditions that predispose to *S. bovis* infection in this population are unclear (15). The purpose of this study was to review the presentation of *S. bovis* bacteremia in pediatric patients. We report the demographic and clinical features of pediatric patients with *S. bovis* bacteremia in a United States–Mexico border city.

## Materials and Methods

Between January 2000 and December 2010, all blood cultures positive for *S. bovis* submitted to the Bacteriologic Reference Laboratory of Texas Tech University Health Science Center, El Paso, TX, USA were identified.

After the approval of the protocol by the Institutional Review Board of Texas Tech University Health Science Center, the patient’s clinical records were systematically reviewed. Using a standardized protocol, we characterized their birth records, infant’s gestational age, birth weight, sex, and age at presentation. Maternal age, antenatal problems, and problems during labor were also identified from infant’s birth records.

The Texas Public Use Data File (PUDF) was used to estimate the total number of deliveries that occurred at the county hospital during the 11-year study period (January 2000 through December 2010). ICD-9-CM code V27 was used to identify deliveries. PUDF data covering the years 2004 through 2007 were readily available to the authors and were used to extrapolate the number of deliveries to the study period. The number of cases of *S. bovis* bacteremia was divided by the estimated number of deliveries at our county hospital.

Concomitant clinical variables were explored. Descriptive statistics of clinical characteristics were conducted by using the means, average, and percentages of data collected. Continuous variables were described using mean and SD, whereas categorical variables were described using frequencies and percentages.

## Results

A total of seven pediatric patients were diagnosed with *S. bovis* bacteremia during the study period. The estimated number of deliveries at the county hospital during the study period was 54,714 and 98% of the babies delivered at UMC were Hispanic, resulting in an unadjusted incidence of *S. bovis* bacteremia of 7/54,714 or 1.3 cases/10,000 births. The proportion of all bacteremias in neonates during that time period due to *S. bovis* was 7/756 (0.93%). If we remove blood cultures positive for *Staphylococcus epidermidis*, a probable contaminant, the clinically relevant proportion would be 7/433 (1.62%).

The neonates with *S. bovis* bacteremia included four (57%) female and three (42%) male, mean age of 1.2 days (range 1–3 days), and all were Hispanic. The clinical features of these babies with *S. bovis* bacteremia are summarized in Table 1. The mothers’ ages ranged from 17 to 33 years. One mother had a prior history of hydrops fetalis. All other obstetrical histories from previous pregnancies were normal. Maternal diagnosis during pregnancy, labor, and delivery included chorioamnionitis (two), oligohydramnios (two), and meconium stained fluid (five). Two had two of these complications. Review of labor and deliveries revealed the following: mode of delivery was spontaneous vaginal delivery (SVD) in five (71%) and caesarian section in two (28%). Six were uncomplicated and one SVD, which had be converted into vacuum assisted. Mean gestational age was 39 weeks (range 38–42); average birth weight was (3.25 kg).

**Table 1:** **Clinical features of neonates with *S. bovis* bacteremia**.

Case No.	Age (days)	Gestational age (weeks)	Sex	Birth weight (kg)	Mode of delivery	Gestational and/or complications after delivery	Treatment (days)
1	1	40	M	3.09	SVD-vacuum assisted	Meconium stained amniotic fluid	Ampicillin – 14
						Respiratory distress	Gentamicin – 7
						Aspiration pneumonia	
2	1	41	M	4.03	SVD	Maternal oligohydramnios	Ampicillin – 10
						Meconium stained amniotic fluid	Cefotaxime – 4
						Respiratory distress	
						Aspiration pneumonia	
						Sepsis	
3	1	39	F	3.25	SVD	Maternal chorioamnionitis	Ampicillin – 10
						Maternal fever in labor	Gentamicin – 10
						Neonatal sepsis	
4	1	40	F	4.06	Caesarian section	Meconium stained amniotic fluid	Ampicillin – 10
						Respiratory distress	
						Sepsis	
5	3	38	M	3.59	SVD	Fever	Ampicillin – 21
						Meningitis	Gentamicin – 21
						Sepsis	
6	1	42	F	3.31	SVD	Maternal chorioamnionitis	Ampicillin – 7
						Meconium stained amniotic fluid	Gentamicin – 7
						Sepsis	
7	1	39	F	2.40	Caesarian section	Meconium stained amniotic fluid	Ampicillin – 10
						Respiratory distress	Gentamicin – 1
						Sepsis	

*SVD, spontaneous vaginal delivery*.

Five out of seven babies presented with abdominal distention and diarrhea. Respiratory distress was present in all babies; all of them had evidence of meconium in the airway based on endotracheal intubation done at birth. Aspiration pneumonia secondary to meconium aspiration was diagnosed in two of them. Clinical evidence of sepsis was present in most of them 6 (75%). All patients except one were treated with combination antibiotics (six), either parenteral ampicillin and gentamicin or ampicillin and cefotaxime. One patient was treated with ampicillin alone. Duration of antibiotic therapy varied from 7 to 21 days (mean 12 days). Endoscopic evaluation was not performed in any of them since it was not clinically indicated. No other complications were noticed at the time of discharge. None of the patients died, but subsequent follow-up was not performed as it was beyond the scope of this study.

## Discussion

In our study of *S. bovis* bacteremia in predominantly Hispanic neonatal patients in a county hospital setting, we found that *S. bovis* is an uncommon pathogen in the newborn that should be identified and treated early to avoid possible substantial morbidity and mortality.

Several cases of *S. bovis* bacteremia have previously been reported in neonates and young infants (14–16). *S. bovis* is considered as an uncommon pathogen in the newborn. This uncommon infection appears to be associated with substantial morbidity and mortality (17). The pathogenesis or clinical conditions that predispose to *S. bovis* infection in this population is unclear. It has been suggested that early-onset (up to 1 week) of *S. bovis* infection might result from transmission of bacteria either intrapartum or during passage through a colonized birth canal and late-onset disease may occur as a consequence of postnatal acquisition of the organism (15).

Different microbiological classification of *S. bovis* subspecies may have various relationships to colorectal neoplasia in adults (8, 18, 19). Patients infected with *S. gallolyticus* subsp *gallolyticus* (biotype I) were noted to have an increased rate of colorectal neoplasia and infective endocarditis (8). In contrast to the *S. bovis* biotype I, *S. bovis* biotype II caused more biliary tract infections and acute cholecystitis than biotype I (18, 19). It would be interesting to known if biotype I was involved in this study but our laboratory did not perform genotype testing.

Since the 1970s, several large series in adults with *S. bovis* bacteremia and its association with colorectal neoplasia has been reported (1, 3, 16, 20). There have been five case series that included both adult and pediatric patients with *S. bovis* bacteremia (10–14). In these previous reported studies, there were 137 adults and only 16 pediatric cases. Other researchers also noted the association of *S. bovis* bacteremia with extra-colonic malignancies, including carcinoma of the esophagus, squamous cell carcinoma of the mouth, adenocarcinoma of the stomach, endometrial cancer, Kaposi sarcoma pancreatic cancer, and gastric lymphoma (11, 18–22). However, these complications have not been reported in children and were not present in our cohort (11, 21–25).

It seems that *S. bovis* bacteremia has different implications in adults and in children. The hypotheses of disease pathophysiology are probably different, as well as the variables of interest. In our series of predominantly Hispanic patients, seven infants were found to have *S. bovis* bacteremia. Contrary to what was described by Gerber et al. (15), all of our patients developed early-onset (<1 week) disease and presented with signs of respiratory distress, which included cyanosis, tachypnea, and apnea attacks. In their case series, most infants presented with gastrointestinal illness with late-onset of disease (>1 week). Five out of seven babies presented with abdominal distention and diarrhea in our series. In both series, it remains uncertain how these babies acquired the infection, but it is possible that early-onset *S. bovis* infection might result from transmission of bacteria either intrapartum or during passage through a colonized birth canal. It has been speculated that gut ischemia from anoxia can compromise the intestinal mucosa allowing translocation of previously commensal bacteria with subsequent systemic dissemination. However, in neonates, respiratory distress is a major sign of neonatal sepsis. Our entire cohort presented with respiratory distress soon after birth, which we could hypothesize to be a sign of an early infection with *S. bovis*, but there was also evidence of meconium in their airway which can also cause respiratory distress in this age group. Another important finding is the presence of meconium stained deliveries as we can speculate the presence of neonatal distress secondary to an early *S. bovis* infection. Late-onset disease may occur as a consequence of postnatal acquisition of the organism (15). It is possible that *S. bovis* may have colonized the meconium (with or without premature rupture of membrane). No other bacteria were reported to grow in blood culture (to support a mixed infection as a cause).

All except one of the babies in our study were treated with combination antibiotics. One was treated with ampicillin alone. All babies received antibiotics for 7–21 days. The response time was not clearly documented. So, we cannot determine how quickly the neonates responded to antibiotic therapy. The most common choice was ampicillin and gentamicin as is the standard of care in the neonatal intensive care unit to treat patients with respiratory distress and suspected sepsis. As reported in a prospective study by Corredoira et al., beta-lactam group of antibiotics are a reasonable first line treatment of *S. bovis* infection given that in their study there was no penicillin resistance among *S. bovis* isolates (26).

Our study has the limitation of being a retrospective study with limited patient size and no control group in a single medical center. This study is limited to infants who developed symptoms after birth and had a blood culture, although there could be an asymptomatic infection that could present later on in life or at another healthcare facility. However, this is one of the few clinical studies in pediatric patients in a predominantly Hispanic population in the United States. Our study was not designed to determine if there were any complications in our patients after their discharge. Specifically, no documented follow-up for meningitis was assessed in our study. At this point, there is no clear evidence of any of the adult complications of *S. bovis* infection for those patients with *S. bovis* bacteremia as newborns. Further studies need to be designed to determine if a neonatal infection will increase the risk of colon cancer as adults.

In conclusion, *S. bovis* bacteremia may be associated with gastrointestinal sepsis with respiratory distress as the most common symptom. In any patient with unexplained *S. bovis* bacteremia, clinicians should be aware of the possibility substantial morbidity and mortality and treat with antibiotics when appropriate.

## Conflict of Interest Statement

The authors declare that the research was conducted in the absence of any commercial or financial relationships that could be construed as a potential conflict of interest.

## Abbreviations

*S. bovis*, *Streptococcus bovis*.
